# Pharmacological and chemogenetic orexin/hypocretin intervention ameliorates Hipp-dependent memory impairment in the A53T mice model of Parkinson’s disease

**DOI:** 10.1186/s13041-019-0514-8

**Published:** 2019-10-30

**Authors:** Milos Stanojlovic, Jean Pierre Pallais, Michael K. Lee, Catherine M. Kotz

**Affiliations:** 1 Integrative Biology and Physiology, University of Minnesota, 2231 6th St SE, Minneapolis, MN 55455 USA; 2 Department of Neuroscience, University of Minnesota, Minneapolis, MN USA; 3 Institute for Translational Neuroscience (ITN), University of Minnesota, Minneapolis, MN USA; 4 Minneapolis VA Health Care System, GRECC, Minneapolis, MN USA

## Abstract

Parkinson’s disease (PD), classically defined as a progressive motor disorder accompanied with dopaminergic neuron loss and presence of Lewy bodies, is the second most common neurodegenerative disease. PD also has various non-classical symptoms, including cognitive impairments. In addition, inflammation and astrogliosis are recognized as an integral part of PD pathology. The hippocampus (Hipp) is a brain region involved in cognition and memory, and the neuropeptide orexin has been shown to enhance learning and memory. Previous studies show impairments in Hipp-dependent memory in a transgenic mouse model of Parkinson’s disease (A53T mice), and we hypothesized that increasing orexin tone will reverse this. To test this, we subjected 3, 5, and 7-month old A53T mice to a Barnes maze and a contextual object recognition test to determine Hipp dependent memory. Inflammation and astrogliosis markers in the Hipp were assessed by immuno-fluorescence densitometry. The data show that early cognitive impairment is coupled with an increase in expression of inflammatory and astrogliosis markers. Next, in two separate experiments, mice were given intra-hippocampal injections of orexin or chemogenetic viral injections of an orexin neuron specific Designer Receptor Exclusively Activated by Designer Drug (DREADD). For the pharmacological approach mice were intracranially treated with orexin A, whereas the chemogenetic approach utilized clozapine N-oxide (CNO). Both pharmacological orexin A intervention as well as chemogenetic activation of orexin neurons ameliorated Hipp-dependent early memory impairment observed in A53T mice. This study implicates orexin in PD-associated cognitive impairment and suggests that exogenous orexin treatment and/or manipulation of endogenous orexin levels may be a potential strategy for addressing early cognitive loss in PD.

## Introduction

Parkinson’s disease is the most prevalent neurodegenerative disease second only to Alzheimer’s [1]. Initially, PD was defined as a motor disorder induced by the loss of dopamine neurons in the substantia nigra pars compacta and the formation of the proteinaceous fibrillar cytoplasmic inclusions called Lewy bodies. Today, mood, cognition, and metabolic impairments are recognized as non-motor symptoms of PD and have been the subject of increasing research in recent decades. Interestingly, many of the non-motor symptoms of PD precede the development of motor disorders [2–5].

Orexin (hypocretin) is a neurotransmitter exclusively produced by orexin neurons located predominantly in the lateral hypothalamus (LH). Major roles for orexin in hypothalamic-regulated physiological functions, including eating behavior, sleep, and spontaneous physical activity [6–11] have been demonstrated, but the complex projection pattern of orexin neurons [12, 13] suggest orexin system involvement in a variety of different processes. Indeed, recent studies indicate that orexins contribute to mood, sleep, cognition, stress, anxiety, and pain regulation [14–20]. It has also been shown that the orexin system is impaired in PD. Reduced levels of orexin in cerebrospinal fluid [21, 22] as well as orexin neuronal loss occur in the late stages of the disease [23, 24], and impairment in orexin circuitry function leads to sleep deficits in PD [25, 26].

The first goal of this study was to determine if the orexin system impairments occurs with the early hippocampus (Hipp) dependent memory deficits in A53T mice. The second goal was to determine if orexin intervention ameliorates A53T-associated changes in Hipp-dependent memory. Hipp-dependent memory assessment was performed using the Barnes maze (BM) assay and the contextual object recognition test (CORT). The BM and CORT are behavioral assays commonly used to measure spatial memory performance and contextual dependent memory, respectively, both of which are associated with Hipp function. For the orexin intervention, we first used intracranial orexin delivery directly to the CA1 region of the Hipp, and then used a chemogenetic approach, to stimulate orexin neurons. Orexin-neuron targeted expression of genetically modified designer receptors exclusively activated by designer drugs (DREADD) was generated using virus containing a DREADD construct encoded in an inverted open reading frame and flanked by lox-p recombination sites, which was stereotaxically injected into the lateral hypothalamus (LH) of orexin-Cre (orx-Cre) and orx-Cre/A53T mice. In orx-Cre mice, Cre-recombinase expression is driven by the Orx promoter, which is exclusive for orexin neurons.

The current studies showed that orexin cell loss was not observed in A53T mice in the early stages of the disease. Finally, both pharmacological and chemogenetic treatment ameliorated cognitive impairments detected in A53T mice. These findings suggest that orexin circuitry dysfunction exists in the A53T mice model of PD and an important role of orexin in Hipp-mediated memory deficits in PD.

## Materials and methods

### Animals and ethics statement

All experimental procedures in this study were approved by the University of Minnesota Animal Care and Use Committee. Mice were maintained on a 12 h light/dark cycle with chow and water ad libitum. Adult male C57BL/6 J (WT), A53T (Hu alpha-Syn (A53T) transgenic line A53T), orx-Cre and orx-Cre/A53T animals were used for this study. The orx-Cre mice were initially obtained from Prof. Takeshi Sakurai (Kanazawa University, JA) and bred on the C57BL/6 J background in our colony. Generation and initial phenotyping of heterozygous orx-Cre and wild type mice was conducted, and has been described previously [27, 28]. The A53T mice were obtained from the Jackson Laboratory (ME, US) and bred on a C57BL/6 J background in our colony or obtained from Professor Michael Lee (University of Minnesota). Heterozygous A53T mice were generated and characterized as described previously [29]. Orx-Cre/A53T mice were generated by crossing orx-Cre positive females and A53T positive males.

### Barnes maze

The Barnes maze test was performed as previously described [30]. The test consists of training (4 days) and test (1 day) trials. The Barnes test exploits the tendency of mice to escape from brightly lit exposed spaces to dark, enclosed spaces. A round platform (100-cm diameter) with 20 holes equally spaced along the periphery was used (San Diego Instruments, CA). The maze was elevated above the floor (100 cm), visual cues were present on the walls of the testing room (within 1–2 m of the maze), and light intensity was set to 200 lx. One hole was connected to an escape box during training, and mice were trained to learn the escape box location over the course of 4 days with 4 trials per day. The maximum length of each training session was 3 min, and the inter-trial interval was 15–20 min. The maze was wiped with 70% EtOH and rotated 90° between each session. Average escape latency was determined daily. A 90 s long probe test was conducted on day 5 with escape box removed. Maze exploration during the probe test was analyzed by calculating time spent in each quadrant (target, + 1, − 1, and opposite). All behaviors were recorded and analyzed using ANY-maze software (San Diego Instruments, CA). All experiments were performed in the morning, between 8 AM and 12 PM.

### Contextual object recognition

The CORT was performed as previously described [31] with slight modifications. The procedure lasts 2 days and consists of 3 phases: day 1 = habituation, day 2 = training + test phase. The animals are able to distinguish between the familiar object in the same context and the familiar object in the non-matching context resulting in more exploratory behavior towards the object that is replaced in the non-matching environment. Two type of enclosures (context A, context B) were used for CORT. Oval, white, opaque, clear enclosure (L x W x H: 32 × 12.5 × 20 cm) and rectangular, white, opaque enclosure with the distinctive visual cues on the walls (L x W x H: 45 × 25 × 20 cm). In the habituation phase (day 1) mice were placed in the box without objects and with no wall cues to freely explore for 10 min. In the training phase (day 2) mice encountered the objects for the first time (two identical objects in a specific context and two other identical objects in another context). The mice are placed in a box (context A) that has no cues on the walls but contain two identical objects for 10 min. After 1 min retention time in the home cage, the mouse is placed for 10 min into another box (context B) with cues on the walls in the form of stripes and which contains two new identical objects. In the test phase (day 3) mice are placed in the context where one of the identical objects is replaced by the other object. For the test phase, the mouse is placed for 10 min in context B containing one object which was present in context B on day 2 and one object which was present in context A on day 2. Discrimination ratio, as a measure for object-in-context recognition memory is calculated as time spent with the novel object compared to the total exploration time of both objects using the following formula: tnovel/(tnovel + tfamiliar).

### Intracranial cannulations and orexin a injections

Mice were anesthetized with an isoflurane mixture (3% for induction, and 1.5% for maintenance), and implanted with bilateral 26-gauge stainless steel cannulae (Plastics One, VA) directed towards the cornus ammonis area-1 (CA1) of dorsal hippocampus (AP: -1.82/DV: -1.7/ML: ±1.3 mm; mm from bregma; 333 nl/5 min) [32]. The injector extended 0.2 mm beyond the tip of the cannula. Animals recovered for 10 days. Prior to artificial cerebrospinal fluid (aCSF)/orexin A injections, animals were acclimated to the restraint and injection procedure with 3 days of artificial aCSF infusion. The infusions of either aCSF 0.2 μl per site (Harvard Apparatus, Holliston, MA USA) or orexin A (American Peptide Co., CA) 250 pmol/0.2 μl per site were made using a syringe pump (KD Scientific Inc., MA) coupled to a Hamilton syringe (Series 7000, Sigma-Aldrich, MO). The animals were gently scuffed to place the bilateral injector into the guide cannula and injectate was delivered into both hemispheres simultaneously over 1 min. All injections occurred between 9 and 11 AM.

### Viral injections

Animals were anesthetized with isofluorane mixture (3% for induction, and 1.5% for maintenance) and placed in a stereotactic apparatus (Kopf Instruments). DREADD targeting was achieved by stereotaxic injection of a Cre-dependent AAV vector expressing a double-floxed inverted open reading frame (DIO) around the DREADD transcript and a fluorescent tag (mCherry). Vectors (AddGene, MA) were injected into the LH (AP: -1.8/DV: -5.5/ML: ±0.9 mm from bregma; 333 nl/5 min) [32] of orx-Cre or orx-Cre/A53T mice. Control groups were injected with pAAV-hSyn-DIO-mCherry (AAV8, 2.1 × 10^13^ GC/ml) (cDREADD). Excitatory neuromodulation was achieved via Gq-coupled pAAV-hSyn-DIO-hM3D (Gq)-mCherry (AAV8, 2.5 × 10^13^ GC/ml) (qDREADD). Animals recovered from the surgery for 4 weeks and were randomly assigned to appropriate experimental groups prior to testing.

### Immunohistochemistry

Mice were perfused intracardially with ice-cold saline, followed by 20 ml of 4% paraformaldehyde (PFA) in PBS (phosphate buffered saline). Brains were harvested and post-fixed in 4% PFA/PBS overnight at 4 °C, followed by 30% (w/v) sucrose in PBS solution at 4 °C until the brains sank. The brains were imbedded in OCT (Optimal Cutting Temperature Compound; Sakura, CA), frozen in dry ice cooled ethanol, and then immediately cut. Coronal brain sections were collected and stored in cryoprotectant (30% (w/v) sucrose, 30% (v/v) Ethylene glycol, 1% (w/v) PVP-40 in PB). Brain sections were washed six times for 5 min with PBS (0.1 m PBS, pH 7.4). After washing sections were incubated with 5% normal horse serum in PBST for 2 hours at room temperature. After washing three times in PBST (0.01% Tween in PBS), the sections were incubated with primary antibodies (mouse anti-p-α-syn (Alpha-synuclein (phospho S129)), Abcam, MA; rabbit anti-GFAP, Abcam, MA; guinea pig anti-IBA1 (anti-ionized calcium binding adaptor molecule 1,), Novus Biologicals, CO; goat anti-orexin A, Santa Cruz, CA; rabbit c-Fos, Santa Cruz, CA; 1:1000) overnight at RT on a platform shaker. Brain sections were washed in PBST four times for ten min after primary antibody incubation and incubated with secondary antibodies conjugated with Alexa Fluor dyes (donkey anti-mouse, donkey anti-rabbit, donkey anti-goat, donkey anti-guinea pig; 1:500, Invitrogen, CA). Brain sections were then washed four times for ten min in PBST and then mounted with ProLong Gold mounting media (Invitrogen, CA).

### Immunofluorescence imaging and image analysis

Immunofluorescence images for GFAP and IBA1 densitometry, and IBA1 positive cell density experiments were captured using the Nikon Eclipse NI-E microscope (Nikon, JA), with a monochrome Nikon Black & White camera DS-QiMc (Nikon, JA). Each fluorochrome is represented as a pseudo-color in the images. For quantification of GFAP and IBA1, every 6th coronal sections from − 1.34 to − 2.30 bregma [32] (four in total) containing CA1 Hipp region were collected and analyzed. Optical density was determined with image analysis software (Image J, National Institutes of Health) by measuring the mean gray value of the CA1 Hipp (20x magnification, two images per area, eight in total). For IBA cell density, Z-stack images (5 μm step) were captured using 20x magnification. The IBA positive cell density in CA1 Hipp region was determined using Image J by counting the positive cells in two areas of the CA1 and of every 6th section (eight in total) and divided by ROI area.

### Unbiased stereology

Unbiased stereology analysis with optical fractionator probe within the Stereo Investigator 11.1.2 software (MBF Bioscience, VT) was used to quantify the number of orexin A positive cell population in LH. Sections were cut at 40 μm to allow for an 18 μm dissector height within each section after dehydration and mounting. Systematic sampling of every 3rd section was collected through the orexin field beginning at bregma − 0.94 and finishing at − 2.18 [32], with the first sampled set of sections chosen at random. Sections were imaged using an Axio Imager M2 fluorescence microscope (Zeiss, DE). Orexin field boundaries were used to outline contours at 5x magnification. Cells were counted using a randomly positioned grid system controlled by Stereo Investigator in a previously defined region in all optical planes. Guard zones were set at 10% of the section thickness to account for damage during the staining procedure. The Grid size was set to 100 × 100 μm and the counting frame to 80 × 80 μm. Counting was performed on 63x magnification (oil). The average coefficient of error (CE, m = 1) ratio for all of the mice imaged was 0.085. On average approximately 260 neurons were counted throughout the entire orexin field of each mouse to give an acceptable coefficient of error (CE) (Gunderson method) of 0.085 using the smoothness factor m = 1. The CE provides a means to estimate sampling precision, which is independent of natural biological variance. As the value approaches 0, the uncertainty in the estimate precision reduces. CE < 0.1 is deemed acceptable within the field of stereology. Cells were only counted if they touched the inclusion border or did not touch the exclusion border of the sampling grid.

### Statistical analyses

All data were analyzed using either Prism 6.0 (GraphPad Software, CA) or SPSS (IBM, NY). Statistical analyses of phenotyping behavioral data were performed using a two-way ANOVA followed by Sidak’s post-hoc analysis. Statistical analyses of pharmacological intervention studies were performed using a one-way ANOVA followed by Tukey’s post-hoc analysis. Statistical analyses of DREADD behavioral data were performed using a one-way ANOVA followed by Tukey’s post-hoc analysis. Unbiased stereology data densitometry and IBA1 cell count data for phenotyping study were analyzed using Student’s T test.

### Experimental design and exclusion criteria

The initial phenotyping study was performed on male 3, 5, and 7-month-old WT and A53T mice. Animal numbers used in the behavioral tests were as follows: BM, *n* = 12/group (3, 5, 7 mo); CORT, 3 mo WT/A53T, *n* = 11/group; 5 mo WT, *n* = 10/group, 5 mo A53T, n = 11/group; 7 mo, WT/A53T, n = 11/group. Three days following the behavioral assays, the animals were sacrificed, and their brains were collected for analysis. Five-month old mice (*n* = 5 per group) were used for IHC analysis. For GFAP and IBA IHC analysis every 6th coronal section containing Hipp from − 1.34 to − 2.30 bregma was collected, stained and analyzed. For the unbiased stereology analysis of orexin neuron numbers, 7-month WT and A53T animals were used. Every third section from − 0.94 to − 2.18 bregma was collected and analyzed using the Stereo Investigator 11.1.2 software. Seven-month WT and A53T animals were used for the unbiased stereology analysis (*n* = 4/group).

For the pharmacological orexin intervention studies 5-month old WT and A53T mice were used. Following a 2-week post-surgery recovery period animals were introduced to the CORT, and observed for performance. Prior to the training phase of the CORT (day 2) mice were injected with either artificial cerebrospinal fluid (aCSF) (WT aCSF; A53T aCSF) or orexin A (WT orexin A; A35T orexin A) and introduced to the CORT (*n* = 9/group).

To test if CNO affects Hipp-dependent memory, 5-month old orx-Cre and orx-Cre/A53T mice were used. Animals were subjected to viral intracranial injections containing cDREADD. Four weeks following surgery animals were introduced to the behavioral CORT. Mice were injected with either saline (orx-Cre cDREADD saline; orx-Cre/A53T cDREADD saline) 30 min prior the behavioral test or CNO (3 mg/kg) dissolved in saline (orx-Cre cDREADD CNO; orx-Cre/A53T cDREADD CNO) 30 min prior the behavioral test (*n* = 6/group).

The chemogenetic study was performed in male 5-month old orx-Cre and orx-Cre/A53T animals given viral intracranial injections containing either cDREADD or qDREADD. After a 4-week recovery period, animals were introduced to the behavioral CORT. Mice were injected with 3 mg/kg of CNO dissolved in saline 30 min prior to the start of the training phase (day 2) (orx-Cre cDREADD CNO; orx-Cre qDREADD CNO; orx-Cre/A53T cDREADD CNO; orx-Cre/A53T qDREADD CNO) (*n* = 10/group).

For orexin and orexin neuronal stimulation effects on inflammation and astrogliosis, 5-month old male animals received either pharmacological injection of orexin A or chemogenetic simulation of the orexin neurons. For the pharmacological study mice received either aCSF (0.2 μl per site) or orexin A (250 pmol/0.2 μl per site) once daily for 10 consecutive days. Experimental groups: WT aCSF, WT orexin A, A53T aCSF, A53T orexin A. For DREADD study, mice received CNO (3 mg/kg) once daily for 10 consecutive days. Experimental groups: orx-Cre cDREAD CNO, orx-Cre qDREAD CNO, orx-Cre/A53T cDREAD CNO, orx-Cre/A53T qDREAD CNO). One day (24 h) following the last treatment animals were sacrificed and every 6th coronal section containing Hipp from − 1.34 to − 2.30 bregma was collected, stained and analyzed (*n* = 5/group).

All animals used in the chemogenetic study were perfused, and their brains were collected for cannulation/injection placement confirmation. Coronal sections containing LH from − 0.94 to − 1.94 bregma were collected and analyzed. Animals were excluded from the experiment if post-hoc histological analyses showed inaccurate cannula/viral injection placement. Mice were observed for neurological deficits and underperformance in behavioral tests. For the pharmacological study a total of 39 mice were cannulated. Three mice were excluded due to neurological issues (lethargy and immobility). For the CNO effects on Hipp dependent cognition study a total of 28 mice were subjected to surgeries. Four mice were excluded due to incorrect viral injection placement. For the effects of chemogenetic stimulation of orexin neurons on Hipp-dependent memory study the total of 51 mice were subjected to surgeries. Three mice were excluded due to neurological issues (lethargy and immobility), while 8 mice were excluded due to incorrect viral injection placement. For DREADD expression confirmation the brains from all animals were analyzed, whereas for c-Fos analyses, only animals with the qDREADD were injected with either saline or CNO (5 mg/kg) 90 min prior to perfusion to confirm functional activation of the DREADD in orexin neurons by c-Fos (immediate early gene) labeling. Every sixth coronal section containing LH from − 0.94 to − 1.94 bregma (*n* = 5 per group) was stained for orexin A and c-Fos and then analyzed and every 6th coronal section containing Hipp from − 1.34 to − 2.30 bregma was stained for c-Fos and then analyzed.

## Results

### Early hippocampus-dependent memory impairment in A53T mice

**Fig. 1:**
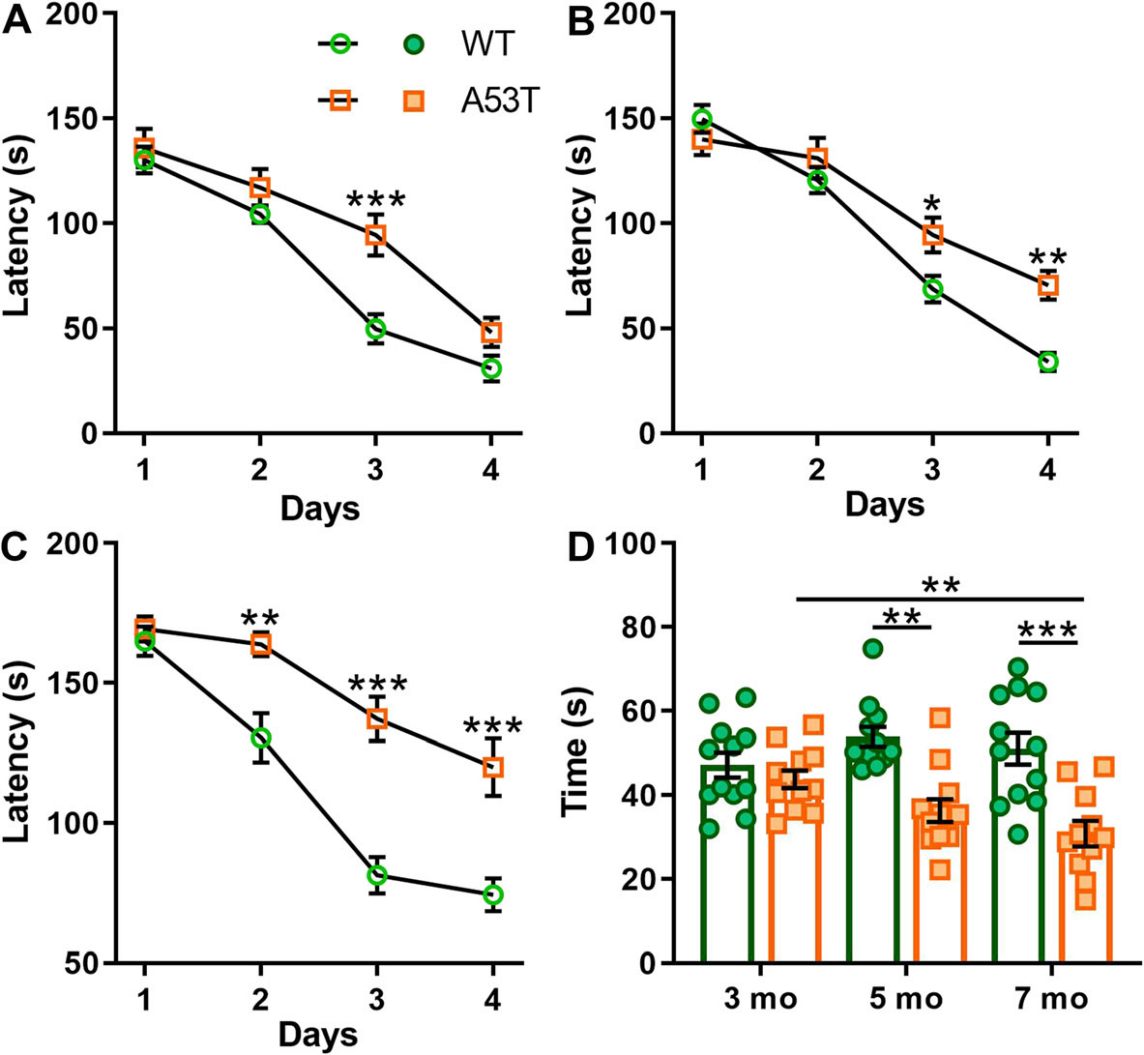
Barnes maze performance for 3, 5, and 7 months old WT and A53T mice. (*A-C*) Latency to escape. In 3-month old mice reduced latency to escape of A53T mice was observed only at day 3 of the training phase (**a**). In 5-month old A53T mice latency to escape was decreased in both day 3 and 4 of the training phase (**b**). In 7-month old A53T mice latency to escape was decreased in training phase days 2, 3 and 4 (**c**). The A53T mice showed reduced time spent in target zone at 5 and 7 months of age compared to WT controls (**d**). Progressive age-dependent reduction of time spent in target zone was observed in A53T mice (**d**). (*n* = 12/group; two-way ANOVA, Sidak; *p < 0.05, **p < 0.01, ***p < 0.005)

**Fig. 2:**
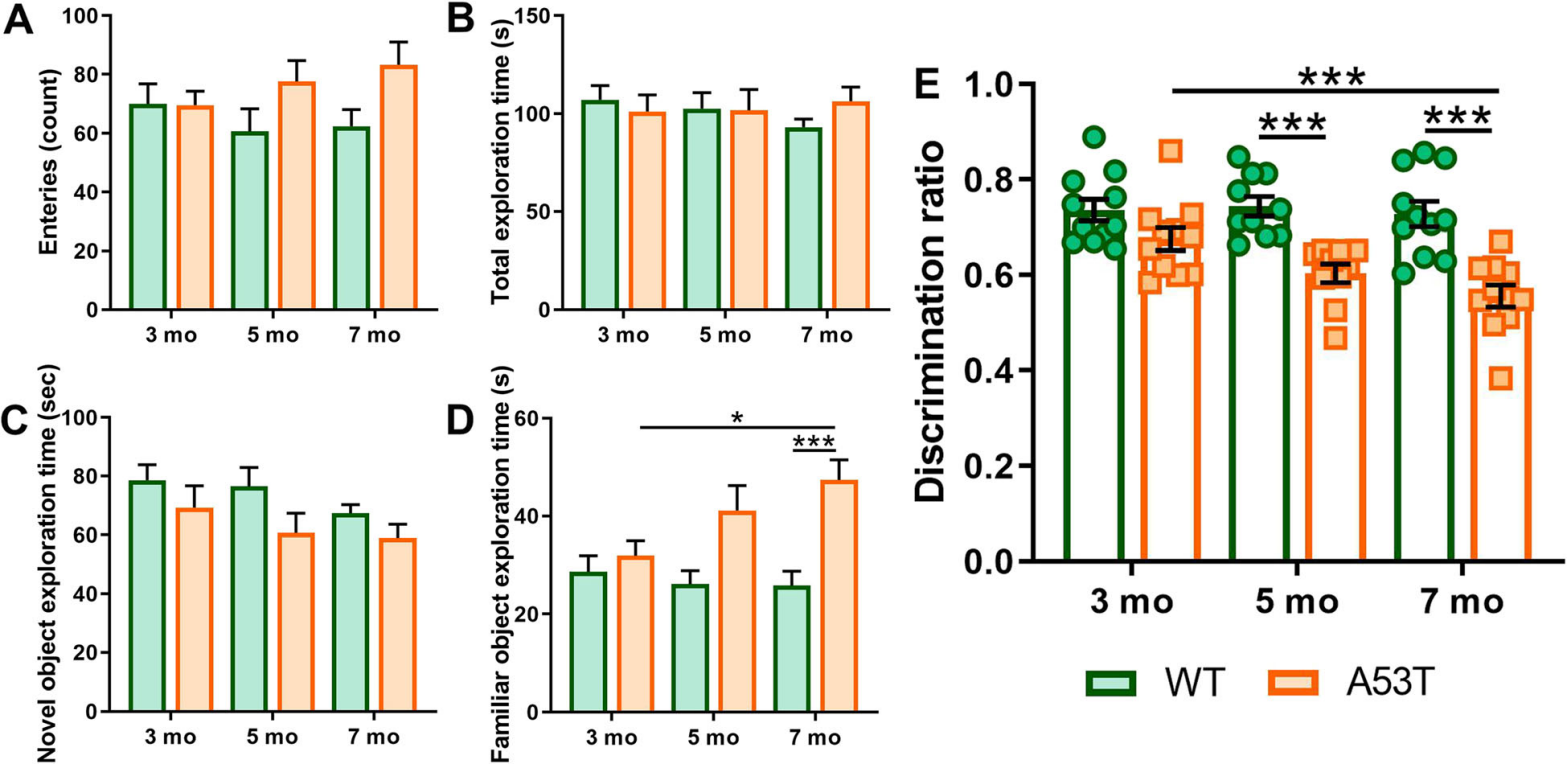
Contextual object recognition test performance for 3, 5, and 7 months old WT and A53T mice. No statistically significant differences were observed in total number of entries in 3, 5 and 7-month old WT and A53T mice (**a**). No statistically significant differences were observed in total exploration time in 3, 5 and 7-month old WT and A53T mice (**b**). No statistically significant differences were observed in novel object exploration time in 3, 5 and 7-month old WT and A53T mice (**c**). Time spend exploring familiar object was increased in 7 mo A53T compared to WT mice (**d**). Age-dependent effects on familiar object exploration time increase was observed in A53T mice (**d**). Decrease in discrimination ratio in A53T mice was observed at 5 and 7 months of age compared to WT mice of the same age (**e**). Age-dependent decrease in discrimination ratio was observed in A53T mice. (3 mo WT/A53T, *n* = 11/group; 5 mo WT, *n* = 10/group; 5 mo A53T, *n* = 11/group; 7 mo WT/A53T, *n* = 11/group; two-way ANOVA, Sidak; *p < 0.05, ***p < 0.005)

To assess Hipp-dependent memory we used 3, 5 and 7-month old WT and A53T mice. Compared to WT controls of the same age A53T mice showed an increased latency to escape in the Barnes maze test on day 3 of the training at 3 months of age (***p < 0.005, Fig. 1a). Increased latency to escape in A53T mice was observed at training days 3 and 4 in 5-month old mice (*p < 0.05, **p < 0.01, Fig. 1b). In 7-month old A53T mice, increased latency to escape was observed at training days 2, 3 and 4 (**p < 0.01, ***p < 0.005, Fig. 1a). Relative to control animals, time spent in the target zone was decreased in 5-month old (**p < 0.01, Fig. 1d) and 7-month old A53T mice (***p < 0.005, Fig. 1d). There was also an age-dependent reduction of time spent in the target zone between 3- and 7-month old A53T mice (**p < 0.01, Fig. 1d), suggesting progressive decline of Hipp-dependent memory task performance. The A53T mice performed similarly in another Hipp-dependent memory task, CORT. Increases in time spent exploring a familiar object was observed between 3 and 7-month old A53T mice (*p < 0.05, Fig. 2c). At 7 months of age A53T mice spent more time investigating a familiar object compared to WT littermates (***p < 0.005, Fig. 2c). Discrimination ratio decreases in the CORT were observed in 5-month old (***p < 0.005, Fig. 2d) and 7-month old A53T mice (***p < 0.005, Fig. 2d) when compared to WT controls. A progressive, age-dependent decrease in discrimination ratio was observed between 3 and 7-month old A53T mice (***p < 0.005, Fig. 2d).

### Densitometry analysis of GFAP and IBA1 expression and IBA1 and orexin a positive cell numbers

**Fig. 3:**
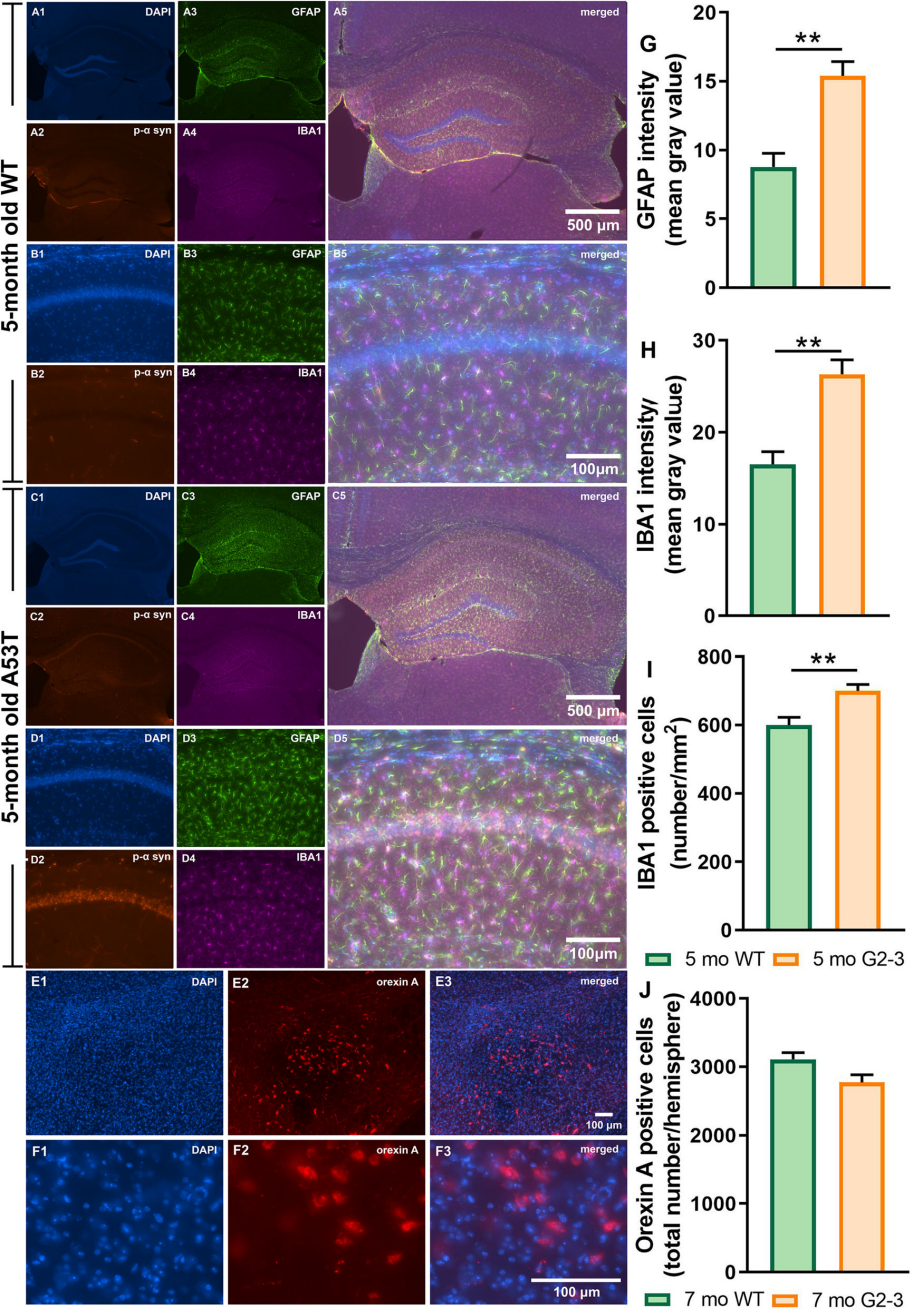
Immunofluorescence (IF) analysis of the hippocampal GFAP and IBA1 expression in CA1 Hipp region and orexin A positive cell numbers in LH of the WT and A53T mice. Representative IF microphotographs of the DAPI, p-α-syn, GFAP, IBA1 and merged image in 5-mo WT mice (**a**, **b**) and A53T mice (**c**, **d**). Representative high-magnification (20x) IF images (**b**, **d**) were used for densitometry analysis. Representative low-magnification (10x) IF images of orexin field (**e**) and high magnification images of orexin neurons (**f**, 63x, oil). Image J was used to quantify the intensity of GFAP and IBA1 staining and density of IBA1 positive cells. The A53T mice showed an increased expression of GFAP (**g**) and IBA1 (**h**) compared to WT mice. The A53T mice showed increased density of IBA1 positive cells (**i**) and no change in orexin A positive cell numbers (**j**). (*n* = 5/group; Student’s T test; **p < 0.01)

The expression of GFAP, a marker of astrogliosis, was increased in A53T mice in the Hipp as compared to their age-matched controls (**p < 0.01; Fig. 3g). An increase in IBA1 expression in the Hipp of the A53T mice was observed (**p < 0.01; Fig. 3h), which was accompanied by increased numbers of IBA1 positive cells (**p < 0.01; Fig. 3i) in the CA1 region of the Hipp. The A53T mice did not show reduced orexin neuron numbers in the LH (Fig. 3j), indicating an absence of orexin neuron loss in A53T mice at 7 months of age.

### Intracranial injection of orexin a and chemogenetic activation of orexin neurons ameliorates hippocampus-dependent memory impairment

**Fig. 4:**
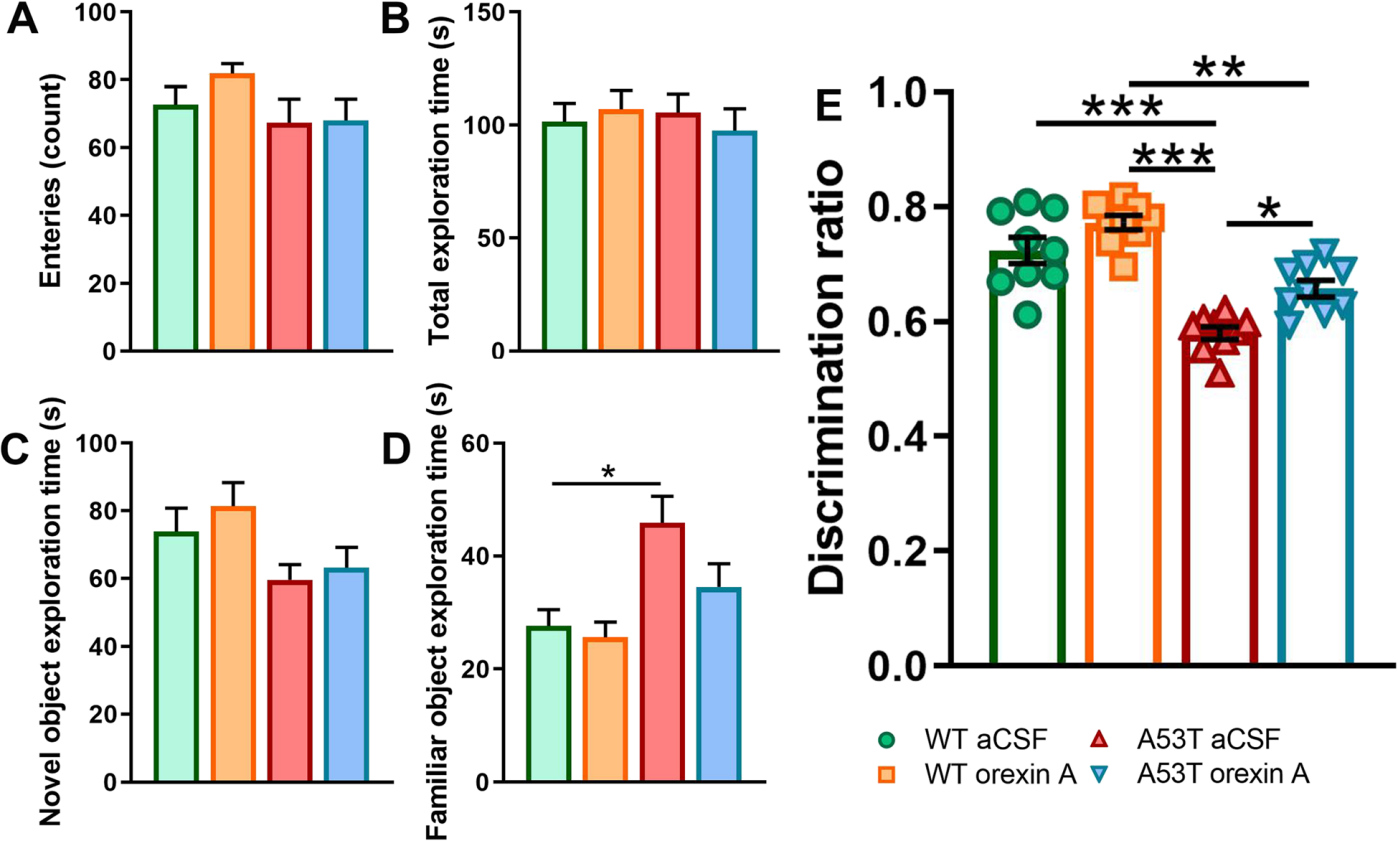
Contextual object recognition test performance for saline and orexin A-treated 5 months old WT and A53T mice. No statistically significant differences were observed in total number of entries between experimental groups (**a**). No statistically significant differences were observed in total exploration time between experimental groups (**b**). No statistically significant differences were observed in novel object exploration time between experimental groups (**c**). Time spend exploring familiar object was increased in WT saline-treated mice compared to WT orexin A-treated (**d**). Decrease in discrimination ratio was observed in A53T saline-treated mice compared to WT saline-treated mice as well as in A53T orexin A-treated mice compared to WT orexin A-treated mice (**e**). Orexin A intervention ameliorated increased discrimination ratio in A53T mice (**e**). Differences in discrimination ratio were observed between WT orexin A-treated mice and A53T saline-treated mice as well. (*n* = 9/group; one-way ANOVA, Tukey’s; *p < 0.05, **p < 0.01, ***p < 0.005)

Compared to WT aCSF-treated mice, A53T aCSF-treated mice showed increased familiar object exploration time of familiar objects (Fig. 4d). Reduced discrimination ratio in CORT was observed in A53T aCSF-treated mice when compared to that in WT aCSF-treated mice, ***p < 0.005, Fig. 4e), and that in WT orexin A-treated animals (***p < 0.005, Fig. 4e). Intracranial orexin A injections increased discrimination ratio in A53T orexin A-treated mice when compared to A53T aCSF-treated mice (**p < 0.01, Fig. 4e). Finally, difference in discrimination ratio in CORT was observed between WT orexin A-treated mice and A53T orexin A-treated mice (**p < 0.01, Fig. 4e).

**Fig. 5:**
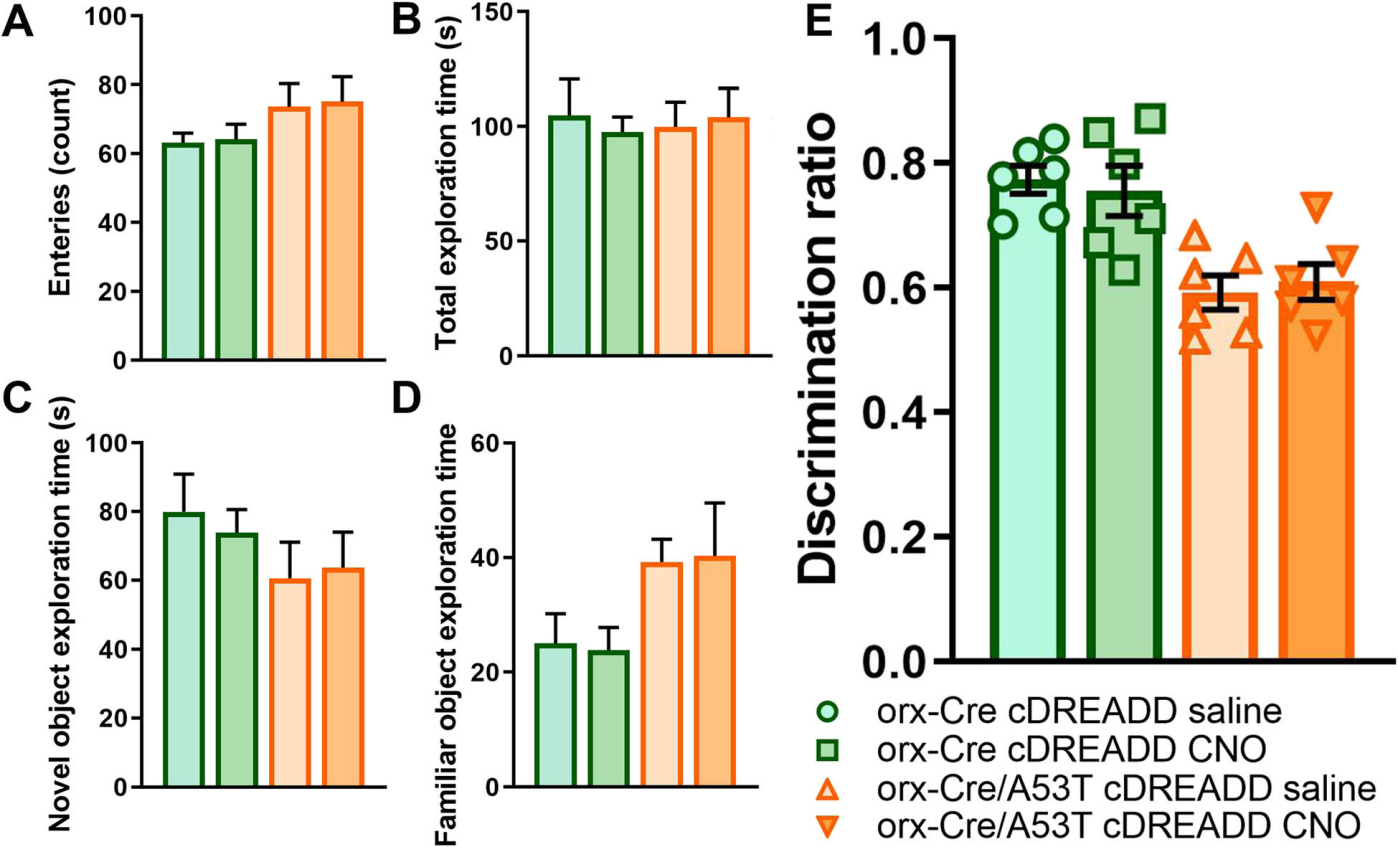
CNO effects on hippocampus-dependent memory in 5-month old orx-Cre and orx-Cre /A53T mice. No statistically significant differences were observed in total number of entries between experimental groups (**a**). No statistically significant differences were observed in total exploration time entries between experimental groups (**b**). No statistically significant differences were observed in novel object exploration time entries between experimental groups (**c**). No statistically significant differences were observed in familiar object exploration time between orx-Cre cDREADD saline vs orx-Cre cDREADD CNO as well as between orx-Cre/A53T cDREADD saline vs orx-Cre/A53T cDREADD CNO (**d**). CNO treatment did not affect discrimination ratio in both orx-Cre and orx-Cre/A53T mice. No statistically significant differences were observed in discrimination retio between orx-Cre cDREADD saline vs orx-Cre cDREADD CNO as well as between orx-Cre/A53T cDREADD saline vs orx-Cre/A53T cDREADD CNO (**e**). (*n* = 6/group; one-way ANOVA, Tukey’s)

Prior to pursuing chemogenetic studies, we addressed a recent report [33] indicating that CNO does not readily cross the blood-brain-barrier in vivo*.* Further, it was reported that CNO converts to clozapine in vivo, which has antipsychotic properties and may affect performance on cognitive-based tasks. Therefore, to exclude the potential independent actions of clozapine in our assay readouts, prior to the DREADD experiment, we performed the CORT in orx-Cre cDREADD and orx-Cre/A53T cDREADD mice to assess if CNO alone affected Hipp-dependent memory. As shown in Fig. 5, there were no effects of CNO on CORT performance, suggesting that the conversion of CNO to clozapine does not affect the outcomes.

**Fig. 6:**
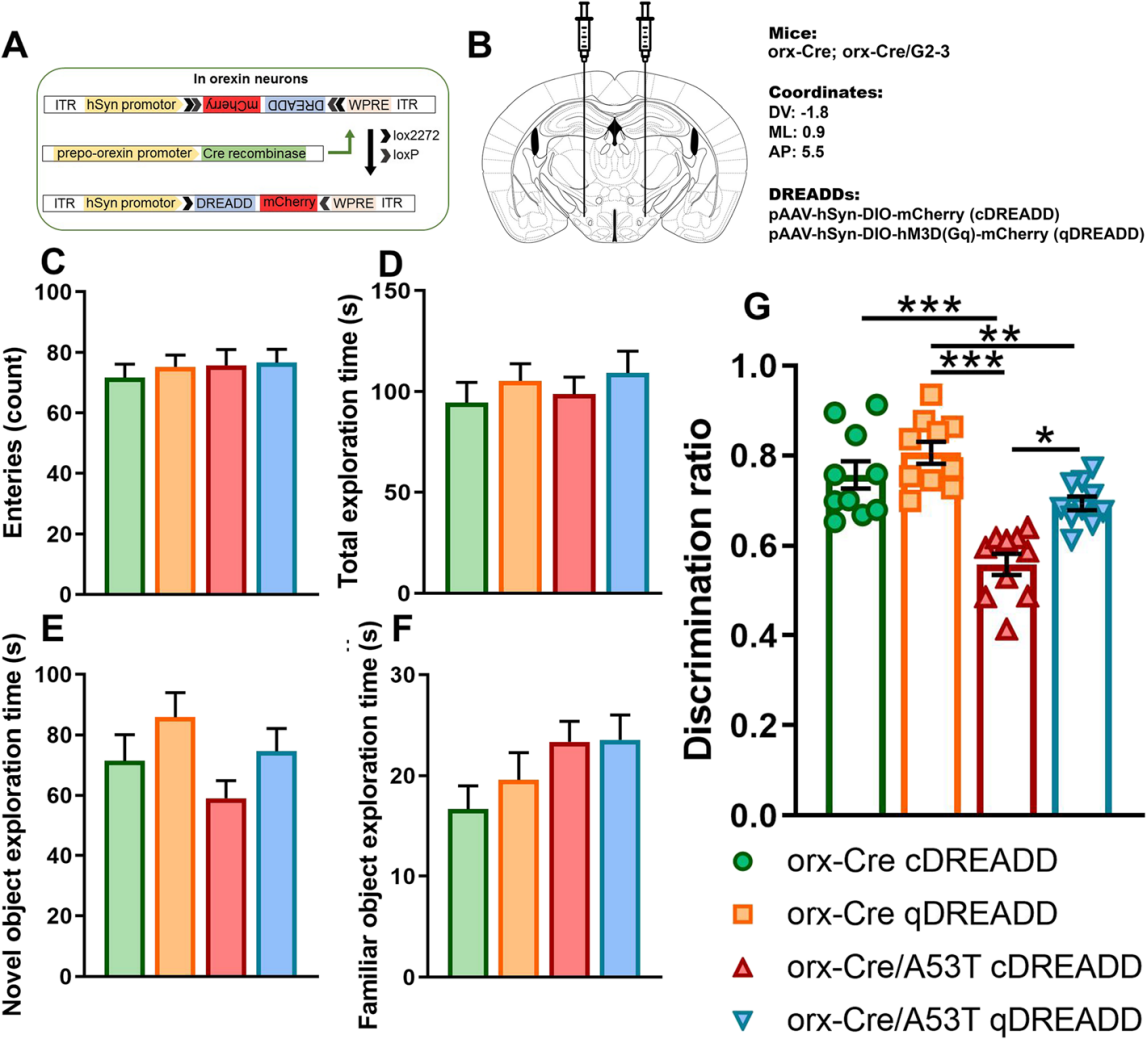
Chemogenetic modulation of hippocampus-dependent memory in 5-month-old orx-Cre/A53T mice. Schematic diagram of AAV vector encoding DREADD-mCherry driven by the human synapsin promoter (hSyn) promoter sequence and flanked by dual flox sites for recombination in the presence of Cre-recombinase (**a**). Cre expression in orx-Cre mice is driven by the prepro-orexin-promoter. Schematic representation of DREADD virus injection site within the lateral hypothalamus (LH) (**b**). DREADD-virus constructs were injected bilaterally (333 nl/5 min). No statistically significant differences were observed in the total number of entries between experimental groups (**c**). No statistically significant differences were observed in total exploration time between experimental groups (**d**). No statistically significant differences were observed in novel object exploration time between experimental groups (**e**). No statistically significant differences were observed in familiar object exploration time between experimental groups (**f**). Reduced discrimination in cDREADD orx-Cre/A53T mice was observed compared to cDREADD orx-Cre mice (**g**). Compared to orx-Cre qDREADD mice, both orx-Cre/A53T cDREADD and orx-Cre/A53T qDREADD showed reductions in discrimination ratio. CNO intervention increased discrimination ratio in orx-Cre/A53T qDREADD mice (**g**) compared to orx-Cre/A53T cDREADD mice. (*n* = 10/group; one-way ANOVA, Tukey’s; *p < 0.05, ***p < 0.005)

To test if chemogenetic orexin neuronal activation modulation can mitigate changes in Hipp-dependent memory in A53T mice, we subjected mice to the CORT. Compared to the orx-Cre cDREADD animals, orx-Cre/A53T c DREADD mice showed a reduced discrimination ratio (orx-Cre cDREADD CNO vs. orx-Cre/A53T cDREADD CNO; ***p < 0.005; Fig. 6g). DREADD induced activation of orexin neurons did not affect the discrimination ratio in orx-Cre mice, but DREADD induced activation of orexin neurons in orx-Cre/A53T mice increased the discrimination ratio (orx-Cre/A53T cDREADD CNO vs. orx-Cre/A53T qDREADD CNO; *p < 0.05) (Fig. 6g). A reduced discrimination ratio was observed in cDREADD orx-Cre/A53T mice as compared to that in cDREADD orx-Cre mice (orx-Cre cDREADD CNO vs. orx-Cre/A53T cDREADD CNO; ***p < 0.005) (Fig. 6g). Compared to orx-Cre qDREADD mice both orx-Cre/A53T cDREADD (orx-Cre qDREADD CNO vs. orx-Cre/A53T cDREADD CNO; ***p < 0.005) and orx-Cre/A53T qDREADD mice (orx-Cre cDREADD CNO vs. orx-Cre/A53T qDREADD CNO; **p < 0.01) showed reduced discrimination ratios (Fig. 6g).

**Fig. 7:**
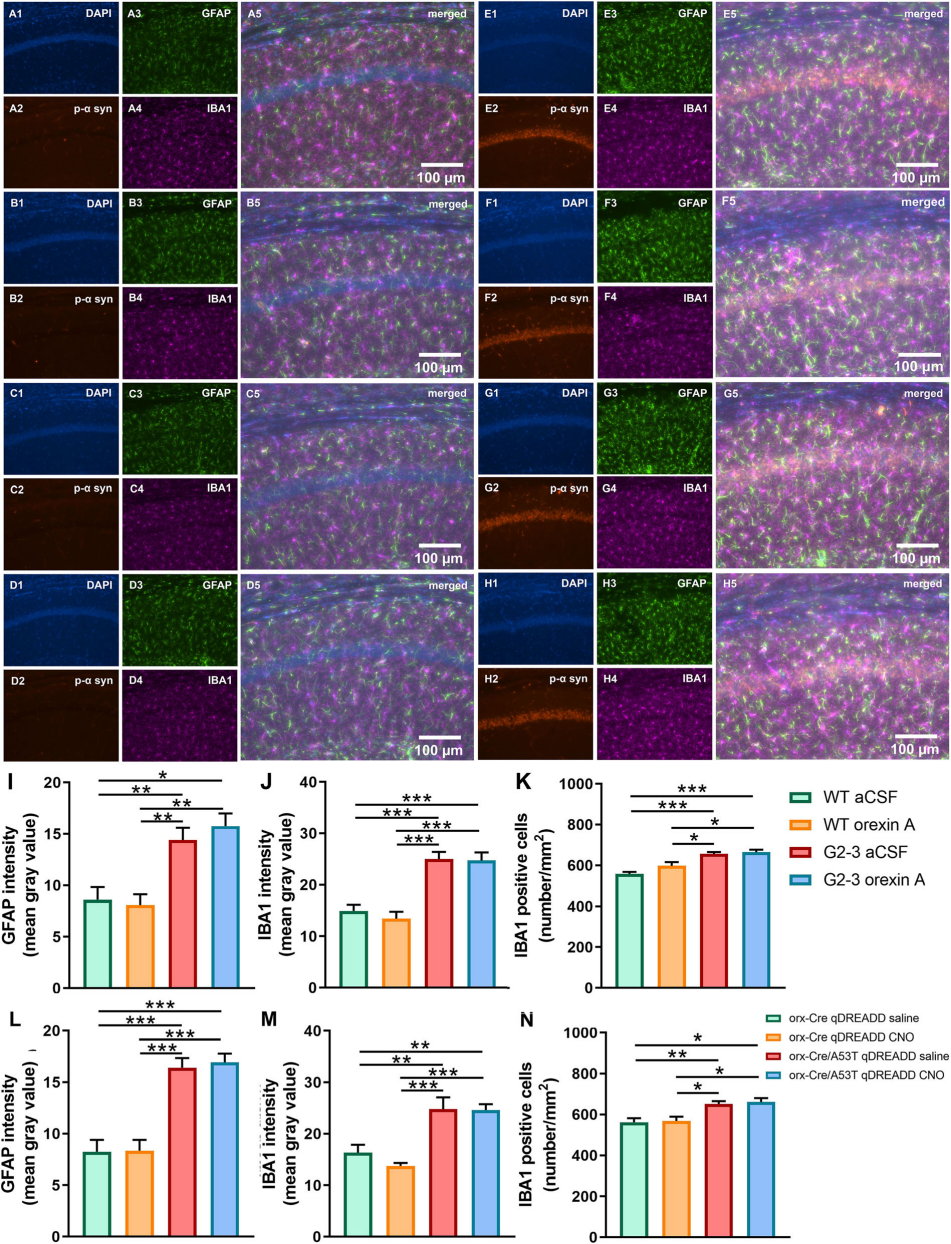
Immunofluorescence (IF) analysis of hippocampal GFAP and IBA1 expression and IBA1 positive cell density in CA1 Hipp region. Representative IF microphotographs of the DAPI, p-α-syn, GFAP, IBA1 and merged image in 5-mo WT aCSF-treated mice (**a**), WT orexin A-treated mice (**b**), A53T aCSF-treated mice (**e**), and A53T orexin A-treated mice (**f**); Representative IF microphotographs of the DAPI, p-α-syn, GFAP, IBA1 and merged image in 5-mo orx-Cre cDREADD CNO-treated mice (**c**), orx-Cre qDREADD CNO (**d**), orx-Cre/A53T cDREADD CNO (**g**), and orx-Cre/A53T qDREADD CNO mice (**h**). Representative high-magnification (20x) IF images (**b**, **d**) were used for densitometry analysis. Image J was used to quantify the intensity of GFAP and IBA1 staining and density of IBA1 positive cells. Neither orexin A nor orexin neuron specific DREADD intervention had any effect on inflammation (**j, k, m, n**) and astrogliosis (**i, l**) (*n* = 5/group; one-way ANOVA, Tukey’s; *p < 0.05, **p < 0.01, ***p < 0.005)

**Fig. 8:**
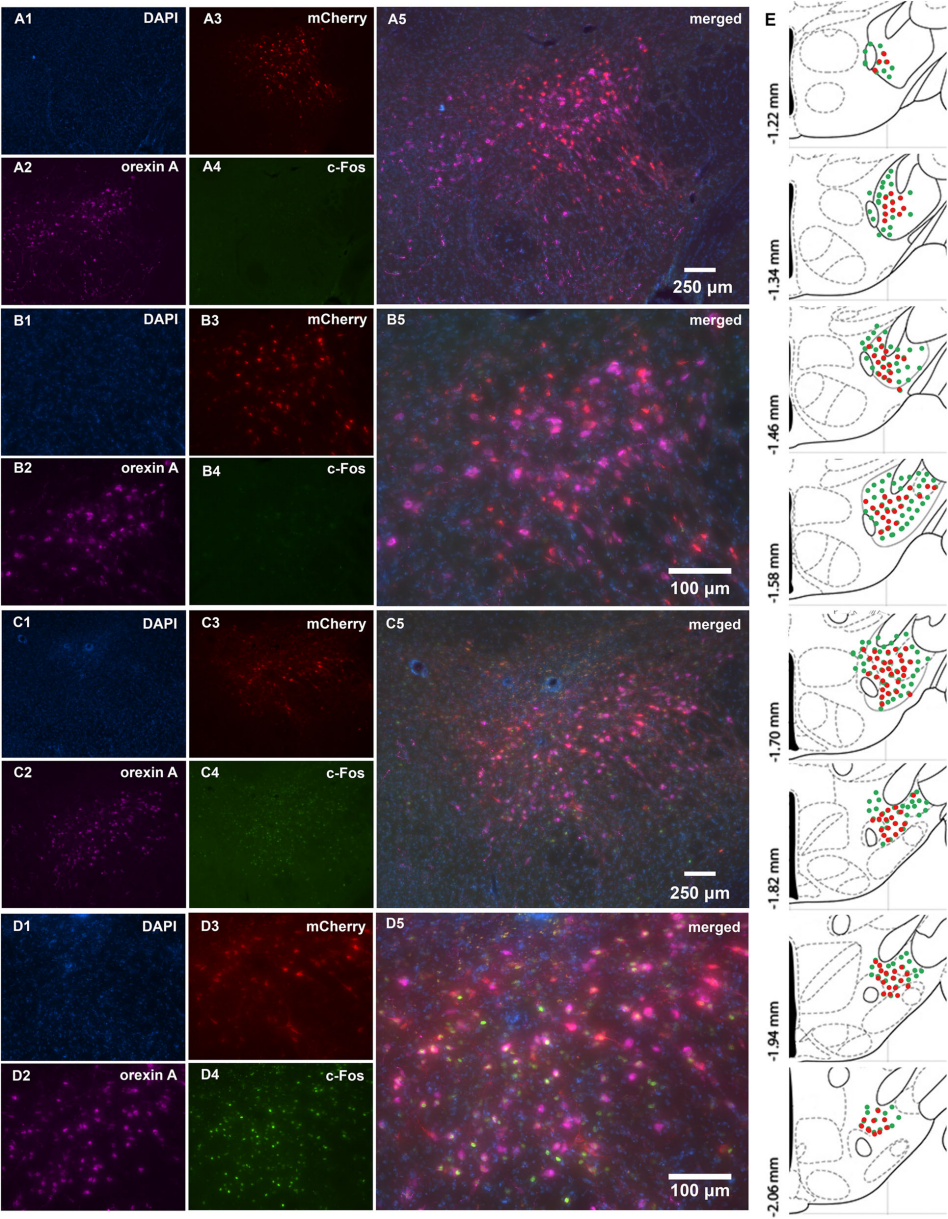
DREADD expression and functionality confirmation. Representative images displaying viral expression of DREADDs, and c-Fos in the lateral hypothalamus (LH) of saline-treated qDREADD mice (**a**, **b**) and CNO-treated qDREADD mice (**c**, **d**). Composite image displaying the spread of viral expression along the LH is depicted using rat brain atlas images [32] (**e**)

### Chronic pharmaceutical orexin a and chemogenetic orexin-specific intervention do not affect inflammation and astrogliosis

For the pharmacological orexin A intervention study, increased GFAP expression was observed in aCSF-treated A53T mice and orexin A-treated A53T mice (WT aCSF vs. A53T aCSF, **p < 0.01; WT aCSF vs. A53T orexin A, **p < 0.01; WT orexin A vs. A53T aCSF, **p < 0.01; WT orexin A vs. A53T orexin A, **p < 0.01)), as compared to WT animals treated with aCSF and WT animals treated with orexin A (Fig. 7i). Increased IBA1 expression was observed in aCSF treated A53T mice and orexin A-treated A53T mice (WT aCSF vs. A53T aCSF, **p < 0.01; WT aCSF vs. A53T orexin A, ***p < 0.005; WT orexin A vs. A53T aCSF, ***p < 0.005; WT orexin A vs. A53T orexin A, ***p < 0.005), as compared to WT animals treated with aCSF and WT animals treated with orexin A (Fig. 7j). When compared to WT animals treated with aCSF and WT animals treated with orexin A increased IBA1 cell density was observed in aCSF-treated A53T mice and orexin A-treated A53T mice (WT aCSF vs. A53T aCSF, ***p < 0.005; aCSF vs. orx-Cre/A53T orexin A, ***p < 0.005; WT orexin A vs. A53T aCSF, *p < 0.05; WT orexin A vs. A53T orexin A, *p < 0.05) (Fig. 7k).

For the orexin neuron stimulation study, increased GFAP expression was observed in orx-Cre/A53T qDREADD saline-treated mice and orx-Cre/A53T qDREADD CNO-treated mice (orx-Cre qDREADD saline vs. orx-Cre/A53T qDREADD saline, ***p < 0.005; orx-Cre qDREADD saline vs. orx-Cre/A53T qDREADD CNO, ***p < 0.005; orx-Cre qDREADD CNO vs. orx-Cre/A53T qDREADD saline, ***p < 0.005; orx-Cre qDREADD CNO vs. orx-Cre/A53T qDREADD CNO, ***p < 0.005) as compared to the orx-Cre qDREADD saline-treated mice and orx-Cre qDREADD CNO-treated mice (Fig. 7l). Increased IBA1 expression was observed in orx-Cre/A53T qDREADD saline-treated mice and orx-Cre/A53T qDREADD CNO-treated mice (orx-Cre qDREADD saline vs. orx-Cre/A53T qDREADD saline, **p < 0.01; orx-Cre qDREADD saline vs. orx-Cre/A53T qDREADD CNO, **p < 0.01; orx-Cre qDREADD CNO vs. orx-Cre/A53T qDREADD saline, ***p < 0.005; orx-Cre qDREADD CNO vs. orx-Cre/A53T qDREADD CNO, ***p < 0.005) as compared to orx-Cre qDREADD saline-treated mice and orx-Cre qDREADD CNO-treated mice (Fig. 7m). Increased IBA1 cell density was observed in orx-Cre/A53T qDREADD saline-treated mice and orx-Cre/A53T qDREADD CNO-treated mice when compared to orx-Cre qDREADD saline-treated mice and orx-Cre qDREADD CNO-treated mice mice (orx-Cre qDREADD saline vs. orx-Cre/A53T qDREADD saline, **p < 0.01; orx-Cre qDREADD saline vs. orx-Cre/A53T qDREADD CNO, *p < 0.05; orx-Cre qDREADD CNO vs. orx-Cre/A53T qDREADD saline, *p < 0.05; orx-Cre qDREADD CNO vs. orx-Cre/A53T qDREADD CNO, *p < 0.05) (Fig. 7n).

### Confirmation of injection placement and DREADD functionality

All of the Orx-Cre/A53T mice used in the DREADD study were screened for orexin A, mCherry and c-Fos expression. Clear co-localization of orexin A and mCherry positive cells were observed in LH. The pattern of c-Fos expression after CNO administration indicated that a majority of orexin neurons responded to CNO treatment. The group of animals that received saline had minimal co-expression of orexin and c-Fos (Fig. 8). Furthermore, CNO treatment induced increase in c-Fos expression in the Hipp of the of the Orx-Cre/A53T mice, while saline did not affect c-Fos levels (n = 5/group; Student’s T test; **p < 0.01) (Additional file 1: Figure S1).

## Discussion

PD is an age-associated disease with a prevalence second only to Alzheimer’s disease. As a result of increased life expectancy due to scientific advancements, the burden of PD is expected to double over the next generation [34]. Since PD predominantly affects older adults, the need to develop strategies to meet the health care needs of individuals with PD is evident [35]. While PD is classified as a movement disorder accompanied by the presence of abnormal protein particles, Lewy bodies, and the loss of dopamine neurons in the substantia nigra, recent studies have established the importance of non-motor symptoms of PD, including cognitive decline [36–39]. In this study, we hypothesized that early cognitive decline in the A53T mouse model of PD can be ameliorated by administration of orexin and by chemogenetic targeting of orexin-neurons.

In the first part of our experiment, we sought to confirm the early age-related Hipp-dependent memory impairment in A53T mice. Further, we tested if cognitive decline is accompanied by inflammation and astrogliosis in Hipp. In A53T mice, non-motor impairments were observed prior to the disease onset [40–43], including progressive memory deficits (Teraviskis et al., 2018, Singh et al., 2019). These mice spontaneously develop the neurodegenerative disease between 9 and 16 months of age with a progressive motoric dysfunction leading to death within 14–21 days of onset (Lee et al., 2002). Overexpression of A53T mutant human α-syn adversely affects neuronal function and increases neuronal toxicity [44, 45]. Inflammation and astrogliosis are contributing factors to PD [46] and A53T-related pathology [47–49]. Furthermore, mutant human α-syn expression in A53T mice is associated with inflammation and astrogliosis [49, 50]. Finally, PD related pathology has been observed in different brain structures of the A53T mice including Hipp and cerebral cortex [51].

Observation of Hipp-dependent memory in A53T mice at 3, 5 and 7 months of age confirmed the presence of early cognitive impairment. This finding agrees with recent studies [52, 53] addressing mechanisms of memory impairment in an A53T mouse model of PD. These studies emphasize the role of Tau protein in synaptic pathology of PD and show that synaptic loss is independent of neurodegeneration. Although differences in the latency to escape in the BM were detected at all observed ages, the time spent in the goal zone was reduced in 5 and 7-month old A53T mice. Mice performed similarly in the CORT. Differences in Hipp-memory dependent tasks were observed in A53T at 5 and 7 months of age. Since both astrogliosis and inflammation are associated with α-syn related pathology [49, 50] as well as with changes in behavior [54–56], it was of interest to determine the potential presence of inflammation and astrogliosis in the Hipp of the A53T mice. Indeed, we observed an increase in IBA1 expression as well as increased IBA1 cell numbers accompanied with increased GFAP expression in the CA1 region of the Hipp.

Due to the complex pattern of orexin neuronal projections, the orexin system is involved in the regulation of diverse functions including cognition and memory. Orexin deficiency results in learning and memory deficits while orexin enhances hippocampal neurogenesis and improves spatial learning and memory [57].. Furthermore, Flores et al. [18] showed that orexin neurons are engaged in the consolidation of fear conditioning and this effect was mainly observed in memories with a high emotional component. Although orexin neurons directly project to amygdala [12, 13], it is believed that the orexin system modulates amygdala-dependent memory indirectly through the locus coeruleus [58]. Orexin release induced by fasting affects conditioned odor aversion learning by increasing both olfactory sensitivity and memory processes underlying the odor-malaise association\ [59]. Finally, a study from Yang et al. [60] showed that orexin neurons contribute to Hipp-dependent memory and synaptic plasticity. In the current study, we did not observe orexin neuronal loss in A53T mice at 5 and 7 months of age. While these results preclude defining a role for orexin in the cognitive losses in young A53T mice, it is well known that synaptic loss and disruptions in neuronal communication precede neurodegenerative processes [61, 62]. Based on this idea, more studies are needed before the possibility of impaired orexin circuitry and function are excluded.

The second part of this study involved modulation of Hipp-dependent memory impairment as well as inflammatory/astrogliosis responses using both pharmacological and chemogenetic approaches. Being primarily perceived as a motor disorder, the diagnosis of PD often relies on the presence of specific motor symptoms. In recent years however, non-motor symptoms are being recognized as an integral part of PD. One of the most common non-motor features is cognitive impairment, which is several times more likely to occur in PD patients than in age-matched controls. Cognitive impairment in PD often precedes the motor signs by many years, which negatively affects quality of life, increased caregiver burden, and annual economic costs [38, 63]. Orexin system function impairment has been observed in PD patients, and early sleep impairments occurring in PD are associated with losses in orexin circuitry function [25, 26]. Furthermore, reduced levels of orexin in cerebrospinal fluid and orexin neuronal losses are detected in the advanced stages of PD [24–26, 64]. Unfortunately, drugs usually used for treatment of PD cause selective loss of orexin neurons. These effects are considered to be due to receptor-mediated presynaptic suppression of glutamatergic excitatory inputs to orexin neurons, leading to chronic silencing of orexin neurons and depletion of orexin [65]. In our experimental setup, intracranial orexin treatment as well as DREADD-mediated activation of orexin neurons improved performance of A53T mice in the CORT, suggesting that loss of orexin circuitry function impacts cognitive functioning in PD, and that this can be mitigated.

Orexin neurons project to many brain regions involved in the regulation of different, seemingly unrelated functions [8, 12, 66]. Based on this wide-spread distribution of orexin signaling, the effects of chemogenetic manipulation of orexin neurons are likely due to a combination of direct effects via orexin neuronal projections to the Hipp (through G-protein coupled orexin receptors) [12, 13, 67], and indirect effects involving different brain regions and accompanying circuitry. One of the structures that mediates indirect orexin effects is the medial septum, as orexin can modulate levels of GABA (gamma-aminobutyric acid) and glutamate neurotransmitters in the Hipp through this structure [68]. Not only does the medial septum play a major role in rhythmogenesis in the Hipp, these two structures interact reciprocally [68]. Additionally, together with areas of prefrontal cortex, ventral striatum, amygdala, and ventral tegmental area, the Hipp is a component of reinforcement circuitry. Orexin neurons project directly to this reinforcement circuit and can affect function, and the Hipp can be modulated by orexin neuronal effects on upstream components of the circuitry [69]. Finally, orexin regulates dopamine signaling in the mesolimbic system [70]. This is particularly interesting for PD-related research given the predominance of dopamine system impairment in this disease [71, 72]. The activation of orexin neurons can consolidate impaired Hipp function through any or all of the above-mentioned means.

Orexin also has a role in inflammatory processes. Activation of orexin receptors activate anti-inflammatory factors such as extracellular-signal-regulated kinases and mitogen-activated phosphate kinase [73, 74]. In addition, pro-inflammatory factors, such as tumor necrosis factor alpha and certain cytokines, impair the function of the orexin system [75]. Inflammation is a strong modulator of behavior and cognition [76–78], but u the current study the orexin interventions did not have any effects on inflammation present in Hipp of the A53T mice, suggesting inflammation-independent mechanisms of orexin action on Hipp-dependent memory.

This study provided several interesting findings. Early Hipp-dependent memory impairment in A53T mice is accompanied by inflammation and astrogliosis. The orexin cell population in A53T mice is not affected in the early stages of the disease. Both pharmacological orexin intervention and orexin neuron-specific chemogenetic intervention ameliorated Hipp-dependent memory impairment. Chronic orexin intervention (both pharmacological and chemogenetic) did not affect inflammation and astrogliosis in the Hipp of the A53T mice. These findings suggest that A53T mice are a model for early cognitive impairment in PD and that orexin plays a role in Hipp-dependent memory in PD. Importantly, the results imply that the orexin system could be a potential target for addressing early cognitive impairment in PD.

## Supplementary information

**Additional file 1:** **Figure S1.** DREADD-mediated activation of orexin neurons affect c-Fos expression in the CA1 region of the Hippocampus (Hipp). Representative DAPI, NeuN, c-Fos and merged images of the Hipp CA1 region of the saline treated qDREADD mice (*A,* 20x; *B,* 40x) and CNO treated qDREADD mice (*C,* 20x; *D,* 40x). Every 6th coronal section containing Hipp from − 1.34 to − 2.30 mm from bregma was stained for c-Fos and then analyzed using image J. Densitometry analysis showed increased expression of c-Fos in the Hipp CA1 region of the CNO treated qDREADD mice compared to saline treated qDREADD controls (*n* = 5/group; Student’s T test; **p < 0.01).

## Data Availability

The datasets during and/or analyzed during the current study available from the corresponding author on reasonable request.

## References

[1] Poewe W, Seppi K, Tanner CM, Halliday GM, Brundin P, Volkmann J (2017). Parkinson disease. Nat Rev Dis Primers.

[2] Anandhan A, Jacome MS, Lei S, Hernandez-Franco P, Pappa A, Panayiotidis MI (2017). Metabolic dysfunction in Parkinson’s disease: bioenergetics, redox homeostasis and central carbon metabolism. Brain Res Bull.

[3] Tan LCS (2012). Mood disorders in Parkinson’s disease. Parkinsonism Relat Disord.

[4] Davis AA, Racette B (2016). Parkinson disease and cognitive impairment. Neurol Clin Pract.

[5] Goldman JG, Litvan I (2011). Mild cognitive impairment in Parkinson’s disease. Minerva Med.

[6] Girault EM, Yi C-X, Fliers E, Kalsbeek A (2012). Orexins, feeding, and energy balance. Prog Brain Res.

[7] Tsujino N, Sakurai T (2009). Orexin/Hypocretin: a neuropeptide at the Interface of sleep, energy homeostasis, and reward system. Pharmacol Rev.

[8] Inutsuka A, Yamanaka A. The physiological role of orexin/hypocretin neurons in the regulation of sleep/wakefulness and neuroendocrine functions. Front Endocrinol. 2013;4 [cited 2017 Mar 18]. Available from: http://journal.frontiersin.org/article/10.3389/fendo.2013.00018/abstract. 10.3389/fendo.2013.00018 PMC3589707 23508038

[9] De Lecea L, Huerta R. Hypocretin (orexin) regulation of sleep-to-wake transitions. Front Pharmacol. 2014;5 [cited 2018 Aug 30]. Available from: https://www.frontiersin.org/articles/10.3389/fphar.2014.00016/full. 10.3389/fphar.2014.00016 PMC3921570 24575043

[10] Kotz CM (2006). Integration of feeding and spontaneous physical activity: role for orexin. Physiol Behav.

[11] Perez-Leighton C, Little MR, Grace M, Billington C, Kotz CM. Orexin signaling in rostral lateral hypothalamus and nucleus accumbens shell in the control of spontaneous physical activity in high- and low-activity rats. Am J Physiol Regul Integr Comp Physiol. 2017;312:R338–46. 10.1152/ajpregu.00339.2016 PMC5401993 28039192

[12] Sakurai T, Nagata R, Yamanaka A, Kawamura H, Tsujino N, Muraki Y (2005). Input of orexin/hypocretin neurons revealed by a genetically encoded tracer in mice. Neuron..

[13] Yoshida K, McCormack S, España RA, Crocker A, Scammell TE (2006). Afferents to the orexin neurons of the rat brain. J Comp Neurol.

[14] Johnson PL, Molosh A, Fitz SD, Truitt WA, Shekhar A (2012). Orexin, stress, and anxiety/panic states. Prog Brain Res.

[15] Yeoh JW, Campbell EJ, James MH, Graham BA, Dayas CV. Orexin antagonists for neuropsychiatric disease: progress and potential pitfalls. Front Neurosci. 2014;8 Available from: https://www.ncbi.nlm.nih.gov/pmc/articles/PMC3934415/. 10.3389/fnins.2014.00036 PMC3934415 24616658

[16] Muschamp JW, Hollander JA, Thompson JL, Voren G, Hassinger LC, Onvani S (2014). Hypocretin (orexin) facilitates reward by attenuating the antireward effects of its cotransmitter dynorphin in ventral tegmental area. PNAS..

[17] Mavanji V, Butterick TA, Duffy CM, Nixon JP, Billington CJ, Kotz CM (2017). Orexin/hypocretin treatment restores hippocampal-dependent memory in orexin-deficient mice. Neurobiol Learn Mem.

[18] Flores Á, Valls-Comamala V, Costa G, Saravia R, Maldonado R, Berrendero F (2014). The Hypocretin/Orexin system mediates the extinction of fear memories. Neuropsychopharmacology..

[19] James MH, Campbell EJ, Dayas CV (2017). Role of the Orexin/Hypocretin system in stress-related psychiatric disorders. Curr Top Behav Neurosci.

[20] Razavi BM, Hosseinzadeh H (2017). A review of the role of orexin system in pain modulation. Biomed Pharmacother.

[21] Wienecke M, Werth E, Poryazova R, Baumann-Vogel H, Bassetti CL, Weller M (2012). Progressive dopamine and hypocretin deficiencies in Parkinson’s disease: is there an impact on sleep and wakefulness?. J Sleep Res.

[22] Drouot X, Moutereau S, Nguyen JP, Lefaucheur JP, Créange A, Remy P (2003). Low levels of ventricular CSF orexin/hypocretin in advanced PD. Neurology..

[23] Fronczek R, Overeem S, Lee SYY, Hegeman IM, van Pelt J, van Duinen SG (2007). Hypocretin (orexin) loss in Parkinson’s disease. Brain..

[24] Thannickal TC, Lai Y-Y, Siegel JM (2007). Hypocretin (orexin) cell loss in Parkinson’s disease. Brain..

[25] Bridoux A, Moutereau S, Covali-Noroc A, Margarit L, Palfi S, Nguyen J-P (2013). Ventricular orexin-a (hypocretin-1) levels correlate with rapid-eye-movement sleep without atonia in Parkinson’s disease. Nat Sci Sleep.

[26] Baumann CR, Scammell TE, Bassetti CL (2008). Parkinson’s disease, sleepiness and hypocretin/orexin. Brain..

[27] Matsuki T, Nomiyama M, Takahira H, Hirashima N, Kunita S, Takahashi S (2009). Selective loss of GABA(B) receptors in orexin-producing neurons results in disrupted sleep/wakefulness architecture. Proc Natl Acad Sci U S A.

[28] Zink AN, Bunney PE, Holm AA, Billington CJ, Kotz CM (2018). Neuromodulation of orexin neurons reduces diet-induced adiposity. Int J Obes (Lond).

[29] Lee MK, Stirling W, Xu Y, Xu X, Qui D, Mandir AS (2002). Human α-synuclein-harboring familial Parkinson’s disease-linked Ala-53 → Thr mutation causes neurodegenerative disease with α-synuclein aggregation in transgenic mice. PNAS..

[30] Victoria NC, de Velasco EMF, Ostrovskaya O, Metzger S, Xia Z, Kotecki L (2016). G protein-gated K+ channel ablation in forebrain pyramidal neurons selectively impairs fear learning. Biol Psychiatry.

[31] Kanatsou S, Kuil LE, Arp M, Oitzl MS, Harris AP, Seckl JR (2015). Overexpression of mineralocorticoid receptors does not affect memory and anxiety-like behavior in female mice. Front Behav Neurosci.

[32] Franklin K (2008). The mouse brain in stereotaxic coordinates.

[33] Gomez JL, Bonaventura J, Lesniak W, Mathews WB, Sysa-Shah P, Rodriguez LA (2017). Chemogenetics revealed: DREADD occupancy and activation via converted clozapine. Science..

[34] Dorsey ER, Constantinescu R, Thompson JP, Biglan KM, Holloway RG, Kieburtz K (2007). Projected number of people with Parkinson disease in the most populous nations, 2005 through 2030. Neurology..

[35] Hirsch L, Jette N, Frolkis A, Steeves T, Pringsheim T (2016). The incidence of Parkinson’s disease: a systematic review and meta-analysis. NED..

[36] Kim I, Shin N-Y, Bak Y, Hyu Lee P, Lee S-K, Mee Lim S. Early-onset mild cognitive impairment in Parkinson’s disease: Altered corticopetal cholinergic network. Sci Rep. 2017;7 [cited 2019 Apr 1]. Available from: https://www.ncbi.nlm.nih.gov/pmc/articles/PMC5443757/. 10.1038/s41598-017-02420-w PMC5443757 28539629

[37] Lawson RA, Yarnall AJ, Duncan GW, Khoo TK, Breen DP, Barker RA (2014). Quality of life and mild cognitive impairment in early Parkinson’s disease: does subtype matter?. J Park Dis.

[38] Watson GS, Leverenz JB (2010). Profile of cognitive impairment in Parkinson disease. Brain Pathol.

[39] Weil RS, Costantini AA, Schrag AE. Mild Cognitive Impairment in Parkinson’s Disease—What Is It? Curr Neurol Neurosci Rep. 2018;18 [cited 2019 Apr 1]. Available from: https://www.ncbi.nlm.nih.gov/pmc/articles/PMC5845587/. 10.1007/s11910-018-0823-9 PMC5845587 29525906

[40] Farrell KF, Krishnamachari S, Villanueva E, Lou H, Alerte TNM, Peet E (2014). Non-motor parkinsonian pathology in aging A53T α-Synuclein mice is associated with progressive synucleinopathy and altered enzymatic function. J Neurochem.

[41] Graham DR, Sidhu A (2010). Mice expressing the A53T mutant form of human alpha-Synuclein exhibit hyperactivity and reduced anxiety-like behavior. J Neurosci Res.

[42] Unger EL, Eve DJ, Perez XA, Reichenbach DK, Xu Y, Lee MK (2006). Locomotor hyperactivity and alterations in dopamine neurotransmission are associated with overexpression of A53T mutant human alpha-synuclein in mice. Neurobiol Dis.

[43] Paumier KL, Rizzo SJS, Berger Z, Chen Y, Gonzales C, Kaftan E (2013). Behavioral characterization of A53T mice reveals early and late stage deficits related to Parkinson’s disease. PLoS One.

[44] Lee S, Oh ST, Jeong HJ, Pak SC, Park H-J, Kim J (2017). MPTP-induced vulnerability of dopamine neurons in A53T α-synuclein overexpressed mice with the potential involvement of DJ-1 downregulation. Korean J Physiol Pharmacol.

[45] Xie Z, Turkson S, Zhuang X (2015). A53T human α-Synuclein overexpression in transgenic mice induces pervasive mitochondria macroautophagy defects preceding dopamine neuron degeneration. J Neurosci.

[46] Phani S, Loike JD, Przedborski S (2012). Neurodegeneration and inflammation in Parkinson’s disease. Parkinsonism Relat Disord.

[47] Booth HDE, Hirst WD, Wade-Martins R (2017). The role of astrocyte dysfunction in Parkinson’s disease pathogenesis. Trends Neurosci.

[48] Fellner L, Jellinger KA, Wenning GK, Stefanova N (2011). Glial dysfunction in the pathogenesis of α-synucleinopathies: emerging concepts. Acta Neuropathol.

[49] Gu X-L, Long C-X, Sun L, Xie C, Lin X, Cai H (2010). Astrocytic expression of Parkinson’s disease-related A53T α-synuclein causes neurodegeneration in mice. Mol Brain.

[50] Du R-H, Zhou Y, Xia M-L, Lu M, Ding J-H, Hu G (2018). α-Synuclein disrupts the anti-inflammatory role of Drd2 via interfering β-arrestin2-TAB1 interaction in astrocytes. J Neuroinflammation.

[51] Tsika E, Moysidou M, Guo J, Cushman M, Gannon P, Sandaltzopoulos R (2010). Distinct region-specific alpha-synuclein oligomers in A53T transgenic mice: implications for neurodegeneration. J Neurosci.

[52] Singh B, Covelo A, Martell-Martínez H, Nanclares C, Sherman MA, Okematti E, et al. Tau is required for progressive synaptic and memory deficits in a transgenic mouse model of α-synucleinopathy. Acta Neuropathol. 2019 [cited 2019 Jul 1]; Available from. 10.1007/s00401-019-02032-w. 10.1007/s00401-019-02032-w PMC6778173 31168644

[53] Teravskis PJ, Covelo A, Miller EC, Singh B, Martell-Martínez HA, Benneyworth MA (2018). A53T mutant alpha-Synuclein induces tau-dependent postsynaptic impairment independently of neurodegenerative changes. J Neurosci.

[54] Kohman RA, Rhodes JS (2013). Neurogenesis, inflammation and behavior. Brain Behav Immun.

[55] Pekny M, Pekna M (2016). Reactive gliosis in the pathogenesis of CNS diseases. Biochim Biophys Acta (BBA) - Mol Basis Dis.

[56] Sofroniew MV, Vinters HV (2010). Astrocytes: biology and pathology. Acta Neuropathol.

[57] Chieffi S, Carotenuto M, Monda V, Valenzano A, Villano I, Precenzano F, et al. Orexin System: The Key for a Healthy Life. Front Physiol. 2017;8 [cited 2019 Apr 9]. Available from: https://www.ncbi.nlm.nih.gov/pmc/articles/PMC5450021/. 10.3389/fphys.2017.00357 PMC5450021 28620314

[58] Sears RM, Fink AE, Wigestrand MB, Farb CR, de Lecea L, LeDoux JE (2013). Orexin/hypocretin system modulates amygdala-dependent threat learning through the locus coeruleus. PNAS..

[59] Ferry B, Duchamp-Viret P (2014). The orexin component of fasting triggers memory processes underlying conditioned food selection in the rat. Learn Mem.

[60] Yang L, Zou B, Xiong X, Pascual C, Xie J, Malik A (2013). Hypocretin/Orexin neurons contribute to Hippocampus-dependent social memory and synaptic plasticity in mice. J Neurosci.

[61] Bae JR, Kim SH (2017). Synapses in neurodegenerative diseases. BMB Rep.

[62] Wishart TM, Parson SH, Gillingwater TH (2006). Synaptic vulnerability in neurodegenerative disease. J Neuropathol Exp Neurol.

[63] Biundo R, Weis L, Antonini A (2016). Cognitive decline in Parkinson’s disease: the complex picture. npj Parkinson’s Dis.

[64] Fronczek R, van Geest S, Frölich M, Overeem S, Roelandse FWC, Lammers GJ (2012). Hypocretin (orexin) loss in Alzheimer’s disease. Neurobiol Aging.

[65] Katsuki H, Michinaga S (2012). Anti-Parkinson drugs and orexin neurons. Vitam Horm.

[66] Tsunematsu T, Yamanaka A (2012). The role of orexin/hypocretin in the central nervous system and peripheral tissues. Vitam Horm.

[67] Farahimanesh S, Karimi S, Haghparast A (2018). Role of orexin-1 receptors in the dorsal hippocampus (CA1 region) in expression and extinction of the morphine-induced conditioned place preference in the rats. Peptides..

[68] Sil’kis IG (2013). Possible mechanisms for the effects of Orexin on hippocampal functioning and spatial learning (analytical review). Neurosci Behav Physiol.

[69] James MH, Mahler SV, Moorman DE, Aston-Jones G (2017). A decade of Orexin/Hypocretin and addiction: where are we now?. Curr Top Behav Neurosci.

[70] Calipari ES, España RA. Hypocretin/orexin regulation of dopamine signaling: implications for reward and reinforcement mechanisms. Front Behav Neurosci. 2012;6 Available from: https://www.ncbi.nlm.nih.gov/pmc/articles/PMC3423791/. 10.3389/fnbeh.2012.00054 PMC3423791 22933994

[71] Surmeier DJ (2018). Determinants of dopaminergic neuron loss in Parkinson’s disease. FEBS J.

[72] Isaias IU, Trujillo P, Summers P, Marotta G, Mainardi L, Pezzoli G, et al. Neuromelanin Imaging and Dopaminergic Loss in Parkinson’s Disease. Front Aging Neurosci. 2016;8 [cited 2019 Jan 30]. Available from: https://www.ncbi.nlm.nih.gov/pmc/articles/PMC4992725/. 10.3389/fnagi.2016.00196 PMC4992725 27597825

[73] Ammoun S, Johansson L, Ekholm ME, Holmqvist T, Danis AS, Korhonen L (2006). OX1 orexin receptors activate extracellular signal-regulated kinase in Chinese hamster ovary cells via multiple mechanisms: the role of Ca2+ influx in OX1 receptor signaling. Mol Endocrinol.

[74] Ramanjaneya M, Conner AC, Chen J, Kumar P, Brown JEP, Jöhren O (2009). Orexin-stimulated MAP kinase cascades are activated through multiple G-protein signalling pathways in human H295R adrenocortical cells: diverse roles for orexins a and B. J Endocrinol.

[75] Zhan S, Cai G-Q, Zheng A, Wang Y, Jia J, Fang H (2011). Tumor necrosis factor-alpha regulates the Hypocretin system via mRNA degradation and ubiquitination. Biochim Biophys Acta.

[76] Castanon N, Luheshi G, Laye S. Role of neuroinflammation in the emotional and cognitive alterations displayed by animal models of obesity. Front Neurosci. 2015;9 [cited 2019 Apr 9]. Available from: https://www.frontiersin.org/articles/10.3389/fnins.2015.00229/full. 10.3389/fnins.2015.00229 PMC4490252 26190966

[77] d’Avila JC, Siqueira LD, Mazeraud A, Azevedo EP, Foguel D, Castro-Faria-Neto HC (2018). Age-related cognitive impairment is associated with long-term neuroinflammation and oxidative stress in a mouse model of episodic systemic inflammation. J Neuroinflammation.

[78] McKim DB, Niraula A, Tarr AJ, Wohleb ES, Sheridan JF, Godbout JP (2016). Neuroinflammatory dynamics underlie memory impairments after repeated social defeat. J Neurosci.

