# Supplementary figures and images for: Pharmacological and chemogenetic orexin/hypocretin intervention ameliorates Hipp-dependent memory impairment in the A53T mice model of Parkinson’s disease
Source: Mol Brain. 2019 Oct 30;12:87. doi: 10.1186/s13041-019-0514-8 (PMC6822428; doi:10.1186/s13041-019-0514-8)

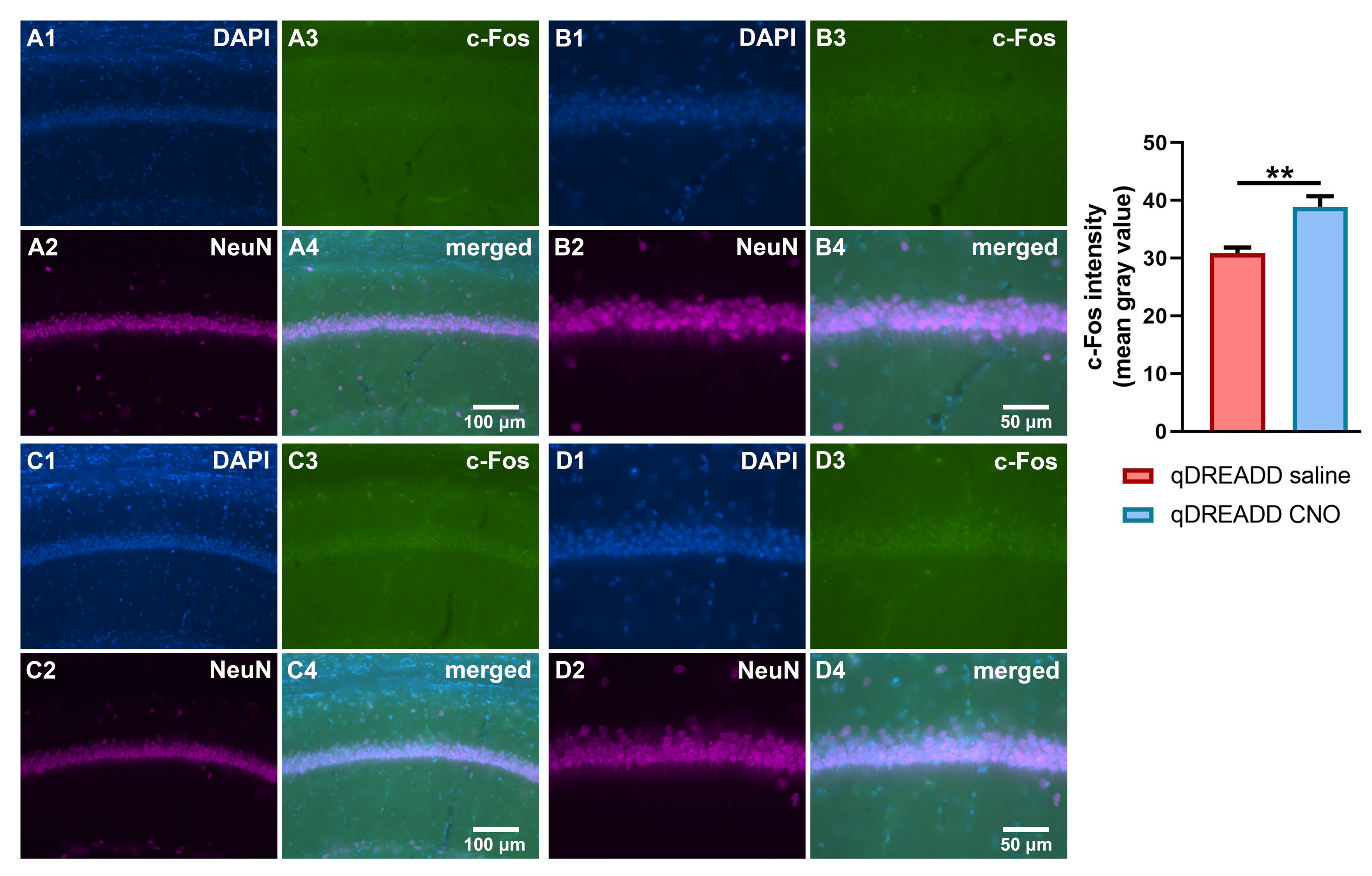

Supplement: Supplementary file 1 — Additional file 1: Figure S1. DREADD-mediated activation of orexin neurons affect c-Fos expression in the CA1 region of the Hippocampus (Hipp). Representative DAPI, NeuN, c-Fos and merged images of the Hipp CA1 region of the saline treated qDREADD mice (A, 20x; B, 40x) and CNO treated qDREADD mice (C, 20x; D, 40x). Every 6th coronal section containing Hipp from − 1.34 to − 2.30 mm from bregma was stained for c-Fos and then analyzed using image J. Densitometry analysis showed increased expression of c-Fos in the Hipp CA1 region of the CNO treated qDREADD mice compared to saline treated qDREADD controls (n = 5/group; Student’s T test; **p < 0.01). [file 13041_2019_514_MOESM1_ESM.tif]
